# Nasogastric tube feeding improves nutritional status and physical state in esophageal cancer patients during chemoradiotherapy: a retrospective study

**DOI:** 10.1007/s00520-023-07780-w

**Published:** 2023-05-17

**Authors:** Shu-an Wang, Wang-shu Dai, Jia-yu Zhu, Bo Gao, Wei Ren, Xiaotian Chen

**Affiliations:** 1 Department of Clinic Nutrition, Nanjing Drum Tower Hospital, The Affiliated Hospital of Nanjing University Medical School, Nanjing, 210008 Jiangsu China; 2 Department of Geriatric Medicine, Nanjing Drum Tower Hospital, The Affiliated Hospital of Nanjing University Medical School, Nanjing, 210008 Jiangsu China; 3 Department of Clinic Nutrition, Gangcheng Rehabilitation Hospital of Zhangjiagang, Suzhou, 215600 Jiangsu China; 4 The Comprehensive Cancer Center of Drum Tower Hospital, Medical School of Nanjing University & Clinical Cancer Institute of Nanjing University, Nanjing, 210008 Jiangsu China

**Keywords:** Esophageal cancer, Malnutrition, Nasogastric tube feeding, Physical state

## Abstract

**Objective:**

To compare the complication rates, nutritional status, and physical state between esophageal cancer (EC) patients managed by nasogastric tube (NGT) feeding versus those managed by oral nutritional supplementation (ONS) during chemoradiotherapy.

**Methods:**

EC patients undergoing chemoradiotherapy managed by nonintravenous nutritional support in our institute were retrospectively recruited and divided into an NGT group and an ONS group based on the nutritional support method. The main outcomes, including complications, nutritional status, and physical state, were compared between groups.

**Results:**

The baseline characteristics of EC patients were comparable. There were no significant differences in the incidence of treatment interruption (13.04% vs. 14.71%, *P* = 0.82), death (2.17% vs. 0.00%, *P* = 0.84), or esophageal fistula (2.17% vs. 1.47%, *P* = 1.00) between the NGT group and ONS group. Body weight loss and decrease in albumin level were significantly lower in the NGT group than in the ONS group (both *P* < 0.05). EC patients in the NGT group had significantly lower Nutritional Risk Screening 2002 (NRS2002) and Patient-Generated Subjective Global Assessment (PG-SGA) scores and significantly higher Karnofsky Performance Status (KPS) scores than patients in the ONS group (all *P* < 0.05). The rates of grade > 2 esophagitis (10.00% vs. 27.59%, *P* = 0.03) and grade > 2 bone marrow suppression (10.00% vs. 32.76%, *P* = 0.01) were significantly lower in the NGT group than in the ONS group. There were no significant differences in the incidence of infection and upper gastrointestinal disorders or therapeutic efficacy between groups (all *P* > 0.05).

**Conclusions:**

EN through NGT feeding leads to significantly better nutritional status and physical state in EC patients during chemoradiotherapy than EN via ONS. NGT may also prevent myelosuppression and esophagitis..

## Introduction

Esophageal cancer (EC) is one of the most common malignancies in China. The incidence of malnutrition in EC patients ranges from 60 to 85%, and it is the leading cause of cancer-associated malnutrition [1]. Malnutrition causes poor sensitivity to both radiotherapy and chemotherapy and increases the incidence of adverse events in EC patients. As a result, the prolonged length of hospital stay and increased medical costs lead to adverse effects on both mental and physical states [2]. Nutritional therapy is an important part of comprehensive anticancer treatment. Clinical evidence has supported the advantage of rational nutritional therapy to increase nutritional reserves, maintain physical fitness and tolerance, reduce complications, promote wound healing, and accelerate a rapid recovery from EC [3]. Enteral nutrition (EN) is preferred for EC patients who have at least partial gastrointestinal function but also have difficulties taking food orally [4]. EN greatly improves nutritional status, reduces complication rates and mortality, and enhances therapeutic efficacy. EN can be classified into tube feeding (TF) and oral nutritional supplementation (ONS), of which the route of TF mainly includes nasogastric (NG) tube (NGT), percutaneous endoscopic gastrostomy (PEG) tube, percutaneous endoscopic jejunostomy (PEJ) tube, and surgical gastrostomy or jejunostomy (SG/SJ) tube. NGT is the most commonly used route of EN tube feeding, and it is characterized by less invasiveness, simple procedures and low cost. Due to poor compliance and substandard energy intake, ONS may provide insufficient nutritional support, which can be avoidable by tube feeding [5, 6]. The current retrospective study compared the complication rate, nutritional status, and physical state of NGT feeding versus ONS in EC patients during chemoradiotherapy, thereby providing references for the selection of optimal EN.

## Patients and methods

### Patients

Clinical data of patients with esophageal cancer diagnosed in the Cancer Center of Nanjing Drum Tower Hospital from December 2018 to April 2021 were retrospectively analyzed. The inclusion criteria were as follows: (1) age > 18 years old; (2) patients histologically or cytologically diagnosed with esophageal squamous cell carcinoma; (3) Nutritional Risk Screening 2002 (NRS2002) score > 3 points; (4) treated with radical concurrent chemoradiotherapy, adjuvant chemoradiotherapy, or palliative chemoradiotherapy; and (5) managed by NGT feeding or ONS. The exclusion criteria were as follows: (1) combined with severe major organ dysfunction, nutritional and metabolic diseases, or autoimmune diseases such as diabetes, hyperlipidemia, and thyroid dysfunction; (2) primary tumors other than EC; (3) stage IV EC patients with distant lymph node metastasis or hematogenous metastasis beyond the region, who were staged using the American Joint Committee on Cancer (AJCC) 8th edition TNM staging criteria [7]; (4) allergy to nutritional preparations; or (5) at the end of life. This study complied with the Declaration of Helsinki and was approved by the Ethics Committee of Nanjing Drum Tower Hospital (approval number: 2018–072-01), and all patients signed informed consent forms.

### Study design

Eligible EC patients were retrospectively recruited and included in the NGT group and ONS group. In this trial, we fully introduced enteral nutrition support methods to patients, including ONS and tube feeding. It was ultimately up to the patients to decide whether to adopt tube feeding or ONS. Notably, in real-world practice, a considerable number of patients in our center are unwilling to tolerate a gastric tube due to discomfort. Complications, nutritional status, and physical state were compared between groups. All patients were clinically diagnosed and treated according to the *Chinese Guidelines for the Diagnosis and Treatment of Esophageal Cancer (2018 Edition)* [8]. The method of radical radiotherapy or palliative radiotherapy in the present study was 95% of the planning target volume (PTV) total dose 60–64 Gy/fractional dose 1.8–2.0 Gy, once a day, 5 times a week. Postoperative adjuvant radiotherapy was performed as follows: (1) R0 postoperative, 95% PTV total dose 50–56 Gy/fractional dose 1.8–2.0 Gy, once a day, 5 times a week; (2) R1/2 postoperative, 95% PTV total dose 50 Gy/divided dose 1.8–2.0 Gy, once a day, 5 times a week. Concurrent chemotherapy during radiotherapy was applied using paclitaxel (45–60 mg/m^2^, d1) + carboplatin (AUC2, d1)/cisplatin (20–25 mg/m^2^, d1)/nedaplatin (20–25 mg/m^2^, d1) every week for 5–6 weeks.

### Nutritional support regimen

According to the *European Society for Parenteral and Enteral Nutrition (ESPEN) guidelines*, a standard energy intake of 25–30 kcal/kg/d was recommended for EC patients during chemoradiotherapy [4]. Ordinary, soft, semiliquid, or liquid food was offered to EC patients depending on their individual condition. They were advised to accept EN when the dietary energy intake was less than 20 kcal/kg/day for more than 1 week. Although the clinical benefits of preventive tube feeding have been highlighted, a considerable number of patients refuse to use it because of the discomfort of intubation. In this retrospective study, patients were fully informed about the procedures of NGT feeding and ONS, and the EN method was ultimately decided by the EC patients themselves.

EC patients in the ONS group received at least 200 g of enteral nutritional powder (ENSURE, 4.5 kcal/g) per day, and those in the NGT group received at least 600 ml of EN suspension (1.5 kcal/ml) per day. Every EC patient was asked to achieve an energy intake of 30 kcal/kg/day. No other micronutrients and vitamins were provided. Nutritional follow-up was performed every week, and individualized nutritional support recommendations were given in a timely manner until the end of the study.

### Data collection

Clinical data of EC patients in both groups were collected before and at the end of chemoradiotherapy, including body weight, body mass index (BMI), serum albumin level (ALB), NRS2002 score, Patient-Generated Subjective Global Assessment (PG-SGA) score, and Karnofsky Performance Status (KPS) score. In addition, treatment interruption, death, esophageal fistula, and infection were recorded. During the course of chemoradiotherapy, the severity of radiation esophagitis, myelosuppression, and upper gastrointestinal disorders such as nausea and vomiting after NGT feeding and ONS were recorded as well.

### Statistical analyses

SPSS 23.0 software (IBM Corp., NY, USA) was used for statistical analyses. Categorical data were compared by the chi-square (χ^2^) test. Fisher’s exact test was performed when one or more expected values were < 5. Normally distributed continuous variables are expressed as the mean ± standard deviation; otherwise, they are expressed as the median and range. Differences in continuous variables were compared by Student’s *t* test or rank sum test.* P* < 0.05 was considered statistically significant at the test level of α = 0.05.

## Results

### Baseline information

**Fig. 1:**
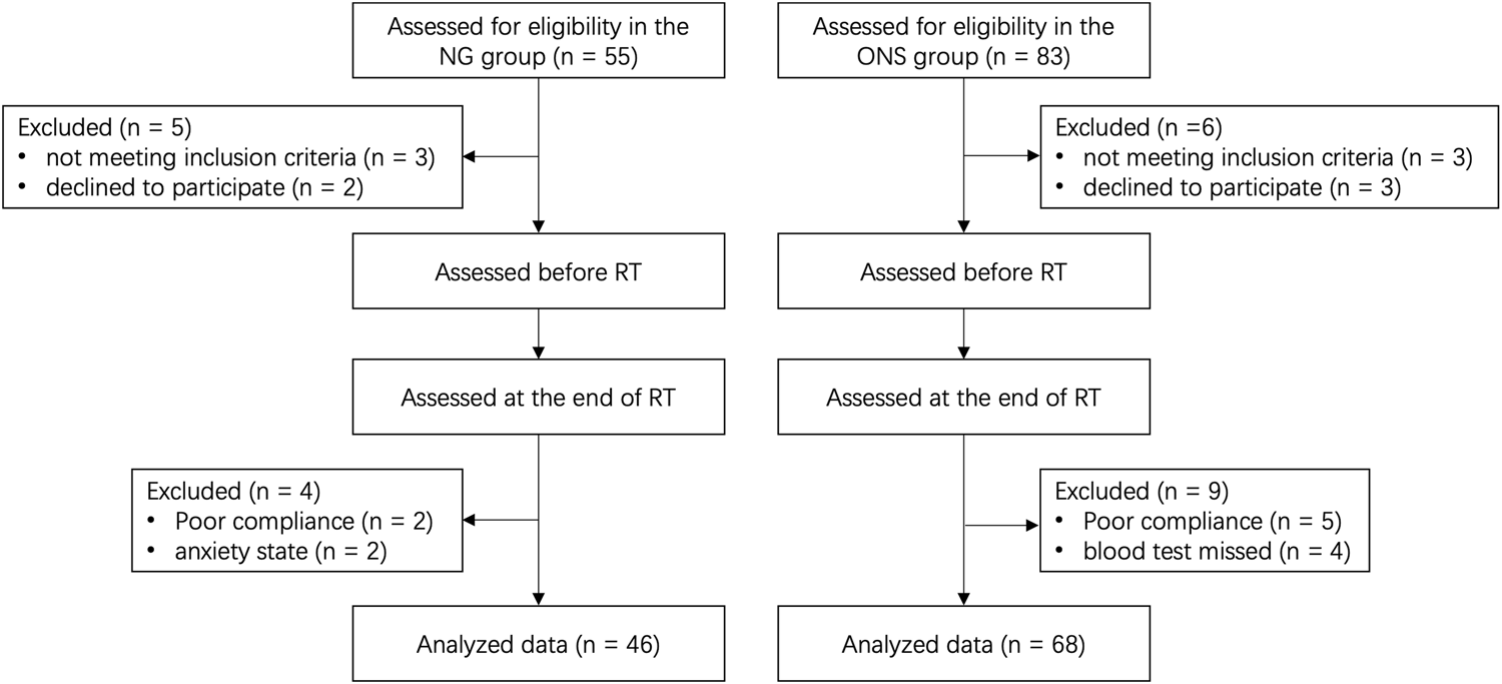
Flow diagram of subject recruitment. *NGT*, nasogastric tube; *ONS*, oral nutritional supplementation; *RT*, radiotherapy

A total of 114 eligible EC patients were retrospectively recruited, all of whom were hospitalized. There were 46 and 68 patients in the NGT group and ONS group, respectively (Fig. 1). There were no significant differences in age, sex, height, weight, BMI, NRS2002, and PG-SGA scores before EN, total energy intake, tumor site, and staging, or treatment methods between groups (all *P* > 0.05). Baseline characteristics of EC patients between the NGT group and ONS group were comparable (Table 1). Most patients started EN in the second week of treatment when they fell victims to radiation esophagitis. They stayed on the EN throughout the treatment course. A total of 26 patients did not finish the treatment course. There were no significant differences in the incomplete rate (13.04% vs. 14.71%, *P* = 0.082) between groups.

**Table 1:** Baseline characteristics of EC patients before enteral nutrition

Variable	NGT group (*n* = 46)	ONS group (*n* = 68)	t/χ^2^	*P* value
Age (year), mean ± SD	67.30 ± 7.12	67.00 ± 8.52	0.19	0.85
Gender (%, *n*)				
Male	80.43 (37/46)	64.71 (44/68)	3.30	0.07
Female	19.57 (9/46)	35.29 (24/68)		
Height (cm), mean ± SD	166.46 ± 6.32	165.22 ± 8.04	0.92	0.36
Weight (kg), mean ± SD	57.36 ± 8.67	59.34 ± 9.57	− 1.12	0.26
BMI (kg/m^2^), mean ± SD	20.67 ± 2.64	21.72 ± 3.12	− 1.87	0.06
NRS2002 score, mean ± SD	3.57 ± 0.62	3.66 ± 0.56	− 0.86	0.39
PG-SGA score, mean ± SD	11.41 ± 2.10	10.85 ± 2.38	1.29	0.20
Grade B	6	16	1.94	0.16
Grade C	40	52		
Total energy intake (kcal·kg^−1^·d^−1^), mean ± SD	1180.57 ± 273.93	1260.95 ± 287.50	− 1.49	0.14
Tumor site (%, *n*)				
Neck	13.04 (6/46)	10.29 (7/68)	0.21	0.65
Thoracic segment	86.96 (40/46)	89.71 (61/68)		
Tumor staging (%, *n*)				
I–II	13.04 (6/46)	22.06 (15/68)	1.48	0.22
III–IV	86.96 (40/46)	77.94 (53/68)		
Treatment (%, *n*)				
Radical chemoradiotherapy	58.70 (27/46)	45.59 (31/68)	− 1.28	0.20
Adjuvant chemoradiotherapy	39.13 (18/46)	52.94 (36/68)		
Palliative chemoradiotherapy	2.17 (1/46)	1.47 (1/68)		

*EC*, esophageal cancer; *BMI*, body mass index; *NRS2002*, Nutritional Risk Screening 2002; *PG-SGA*, Patient-Generated Subjective Global Assessment; *SD*, standard deviation; *NGT*, nasogastric tube; *ONS*, oral nutritional supplementation

### Adverse events

There were no significant differences in the incidences of treatment interruption, death, and esophageal fistula between groups (all *P* > 0.05, Table 2).

**Table 2:** Adverse events of EC patients managed by NGT feeding and ONS

Adverse event (%, *n*)	NGT group (*n* = 46)	ONS group (*n* = 68)	χ^2^	*P* value
Treatment interruption	13.04 (6/46)	14.71 (10/68)	0.06	0.82
Death	2.17 (1/46)	0.00 (0/68)	0.04	0.84
Esophageal fistula	2.17 (1/46)	1.47 (1/68)	0.08	1.00

*EC*, esophageal cancer; *NGT*, nasogastric tube; *ONS*, oral nutritional supplementation

### Evaluation of nutritional status and physical state

**Fig. 2:**
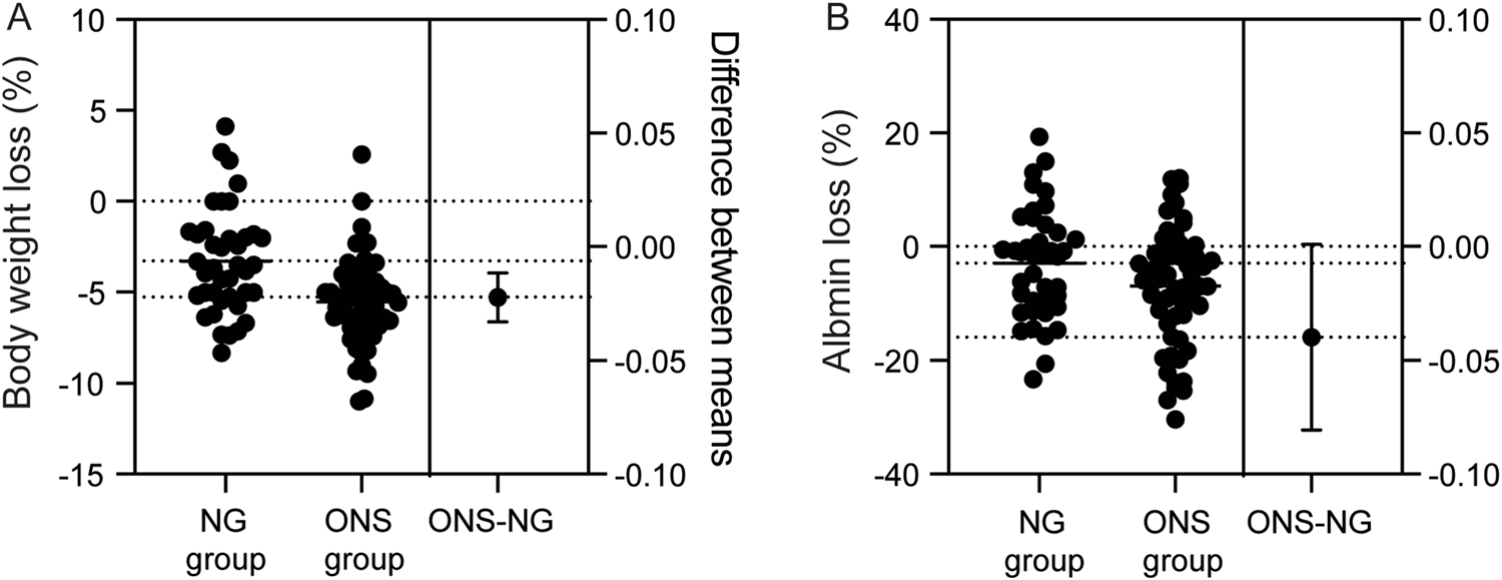
Estimation plot of body weight loss (**A**) and decrease in albumin level (**B**) between NGT feeding and ONS group. *NGT*, nasogastric tube; *ONS*, oral nutritional supplementation

A total of 6 and 10 EC patients in the NGT group and ONS group, respectively, were excluded for treatment interruption. The ALB level in the NGT group was significantly higher than that in the ONS group (37.57 ± 2.86 g/L vs. 35.84 ± 4.46 g/L, *P* = 0.02). No significant difference was detected in body weight (55.57 ± 8.07 kg vs. 56.81 ± 8.67 kg, *P* = 0.48). The body weight loss (Fig. 2A) and decrease in ALB level (Fig. 2B) after EN were significantly larger in the ONS group than in the NGT group. Before EN, NRS2002 scores (3.65 ± 0.62 vs. 3.66 ± 0.58, *P* = 0.97), PG-SGA scores (11.50 ± 2.22 vs. 10.93 ± 2.53, *P* = 0.25), and KPS scores (87.75 ± 8.91 vs. 90.17 ± 7.37, *P* = 0.15) were comparable between groups. Notably, EC patients in the NGT group had significantly lower nutritional NRS2002 (1.90 ± 0.50 vs. 2.43 ± 0.70, *P* < 0.001) and PG-SGA scores (7.50 ± 1.92 vs. 8.53 ± 2.64, *P* = 0.03) and significantly higher KPS scores (65.25 ± 5.06 vs. 61.55 ± 5.23, *P* < 0.001) than patients in the ONS group, thus suggesting that NGT feeding effectively improved the nutritional status and physical state in EC patients (Table 3).

**Table 3:** Nutritional status and physical state of EC patients managed by NGT feeding and ONS

Variable		NGT group (*n* = 40)	ONS group (*n* = 58)	t	*P* value
NRS2002 score, mean ± SD	Before EN	3.65 ± 0.62	3.66 ± 0.58	− 0.04	0.97
	After EN	1.90 ± 0.50	2.43 ± 0.70	− 4.38	0.00*
PG-SGA score, mean ± SD	Before EN	11.50 ± 2.22	10.93 ± 2.53	1.15	0.25
	After EN	7.50 ± 1.92	8.53 ± 2.64	− 2.24	0.03*
PG-SGA grade, Grade B/C	Before EN	6/34	15/43	1.66	0.20
	After EN	28/12	30/28	3.3	0.07
KPS score, mean ± SD	Before EN	87.75 ± 8.91	90.17 ± 7.37	− 1.45	0.15
	After EN	65.25 ± 5.06	61.55 ± 5.23	3.51	0.00*

*EC*, esophageal cancer; *EN*, enteral nutrition; *NRS2002*, Nutritional Risk Screening 2002; *PG-SGA*, Patient-Generated Subjective Global Assessment; *KPS*, Karnofsky Performance Status; *SD*, standard deviation; *NGT*, nasogastric tube; *ONS*, oral nutritional supplementation; *, for *P* < 0.05

### Complications and efficacy

The incidences of grade > 2 esophagitis (10.00% vs. 27.59%, *P* = 0.03) and myelosuppression (10.00% vs. 32.76%, *P* = 0.01) were significantly lower in the NGT group than in the ONS group. There were no significant differences in the incidences of infection (20.00% vs. 6.90%, *P* = 0.10) and upper gastrointestinal disorders (0.00% vs. 5.17%, *P* = 0.39), as well as the efficacy rate of partial response (PR) + complete response (CR) (100% vs. 91.38%, *P* = 0.15) between groups (Table 4).

**Table 4:** Complications and therapeutic efficacy of EC patients managed by NGT feeding and ONS

Variable (%, *n*)	NGT group (*n* = 40)	ONS group (*n* = 58)	χ^2^	*P* value
Complications				
Infection	20.00 (8/40)	6.90 (4/58)	2.66	0.10
Esophagitis				
Grade ≤ 2	90.00 (36/40)	72.41 (42/58)	4.51	0.03^*^
Grade ≥ 3	10.00 (4/40)	27.59 (16/58)		
Myelosuppression				
Grade ≤ 2	90.00 (36/40)	67.24 (39/58)	6.83	0.01^*^
Grade ≥ 3	10.00 (4/40)	32.76 (19/58)		
Upper gastrointestinal disorders				
Grade ≤ 2	100.00 (40/40)	94.83 (55/58)	0.75	0.39
Grade ≥ 3	0.00 (0/40)	5.17 (3/58)		
Therapeutic efficacy				
PD + SD	0.00 (0/40)	8.62 (5/58)	2.07	0.15
PR + CR	100.00 (40/40)	91.38 (53/58)		

*EC*, esophageal cancer; *PD*, progressive disease; *SD*, stable disease; *PR*, partial response; *CR*, complete response; *RTOG*, Radiation Therapy Oncology Group; *NGT*, nasogastric tube; *ONS*, oral nutritional supplementation; ^*^, for *P* < 0.05

## Discussion

Concurrent chemoradiotherapy is an important therapeutic strategy for middle- or advanced-stage EC patients [8]. Malnutrition is the most common complication in EC patients and may lead to poor prognosis and even death. EN has been widely applied to EC patients with the development of nutrition theory in recent years. The present study showed that NGT feeding contributed to improving the nutritional status and physical state during chemoradiotherapy in EC patients, as well as alleviating relevant complications.

Clinical evidence has validated that TF provides clinical benefits to EC patients by maintaining body weight and nutritional status, reducing toxicity, preventing treatment interruption, improving physical state, and shortening the length of hospital stay [9–11]. It has been reported that TF significantly reduces the incidence of skeletal muscle loss, grade ≥ 2 myelosuppression, and myelosuppression-related fever in locally advanced EC patients treated with radical chemoradiotherapy [10]. Patients with esophageal fistula also benefit from EN. A retrospective study involving 40 patients with esophageal fistula showed that TF effectively promotes fistula closure, enhances tolerance, and prolongs overall survival [6]. Our study consistently discovered that the incidences of grade ≥ 3 esophagitis (10.00% vs. 27.59%, *P* = 0.03) and grade ≥ 3 myelosuppression (10.00% vs. 32.76%, *P* = 0.01) in the NGT group were significantly lower than those in the ONS group. However, the incidence of upper gastrointestinal disorders and therapeutic efficacy were comparable between groups.

Han et al. [12] revealed that up to 58.7% of EC patients have a significantly insufficient energy intake during radiotherapy, with an average weight loss of 2.42 ± 2.4 kg. In the present study, EC patients in the NGT group and ONS group lost an average weight of 1.95 kg and 3.40 kg, respectively. Weight loss of more than 5 kg is considered a negative hallmark for malignancies [8]. Dong et al. [13] found that weight loss of more than 10% is significantly associated with grade ≥ 2 radiation esophagitis. Moreover, weight loss often manifests as skeletal muscle loss [14], which is an important predictor of overall survival and recurrence-free survival in EC patients [15]. ALB is also a critical indicator. Dong et al. [13] illustrated that ALB < 35 g/L is associated with moderate or severe radiation esophagitis. ALB < 30 g/L is considered a predictor for an increased risk of postoperative anastomotic leakage [8]. Our data found that weight loss and decrease in ALB level after EN were significantly more pronounced in the ONS group than in the NGT group.

The NRS2002 score is a simple and practical predictor of clinical outcomes and is recommended as a nutritional risk screening tool for EC patients [16, 17]. The PG-SGA was first proposed by American scholar Ottery in 1994 and was developed based on subjective global assessment (SGA) [18]. It is specifically designed for assessing the nutritional status of cancer patients, the efficacy of which has been validated by the American Dietetic Association (ADA) and other academic groups [19, 20]. Movahed et al. [21] conducted a cross-sectional study involving 189 newly diagnosed patients with EC who were assessed by PG-SGA. They found that 79% of EC patients have a PG-SGA score greater than 8, thus suggesting the need for nutritional intervention and symptom management. The PG-SGA score is significantly correlated with nutritional status and prognosis [22–24]. The present study discovered that EC patients in the NGT group had significantly lower NRS 2002 and PG-SGA scores after EN than those in the ONS group, indicating that NGT feeding potentially provided a better nutritional status and a better prognosis.

Weight loss and ALB reduction, although unavoidable during radiotherapy for EC patients, can be effectively alleviated by TF [25]. Since TF is independent of active feeding in EC patients, it is easier to achieve the target energy intake. Dong et al. [6] demonstrated that the average energy intake of EC patients by TF is up to 2166 kcal/d. TF is recommended as a nutritional supplemental route to EC patients with moderate or severe complications that affect oral feeding. According to the length of intubation, NG, PEG/PEJ, or SG/SJ is recommended based on the individualized condition. If TF is not provided in a timely manner, serious complications can occur, such as mucosal erosion, ulceration, bleeding, catheter prolapse, or blockage [26]. In clinical practice, NGT feeding is given priority for its advantages, such as a low risk of trauma, regardless of the long-term requirement for nutritional support. Potential adverse events due to long-term TF should be considered.

There were several limitations in the present study. First, it was a retrospective study that caused some biases, and our conclusion needs to be validated in randomized controlled trials in the future. Second, subjects were recruited in a single center, and large-scale multicenter studies are required to enhance the reliability. Third, we did not record calorie consumption data in this trial, so the difference in calorie consumption between the groups is unknown. Finally, complications, nutritional status, and physical state in EC patients were compared during the chemoradiotherapy stage, and the benefits of NGT feeding should be explored across long-term follow-up visits.

The current study used a retrospective design, and clinical data of eligible patients were obtained after obtaining permission by contacting them through telephone. EC patients with poor compliance were given parenteral nutrition supplementation rather than forced to continue ONS or TF. Only a few patients did not comply with their interventions, which would not influence the final conclusion.

In conclusion, NGT feeding may be more effective in improving the nutritional status and physical state and reducing relevant complications than ONS feeding among EC patients during chemoradiotherapy.

## Data Availability

The data are available from the corresponding author on reasonable request.
